# Supplementary material for: C-Terminal Domain of the Human Zinc Transporter hZnT8 Is Structurally Indistinguishable from Its Disease Risk Variant (R325W)
Source: Int J Mol Sci. 2020 Jan 31;21(3):926. doi: 10.3390/ijms21030926 (PMC7037036; doi:10.3390/ijms21030926)
Supplement: Supplementary file 1 [file ijms-21-00926-s001.pdf]

Supplementary Materials

Supplementary Figures:

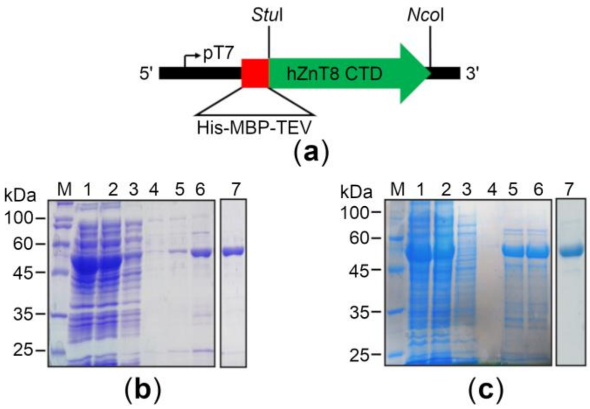

Figure S1. Strategy for improved expression of the C-terminal domains (CTDs) of hZnT8 (the wild-type (WT) and the R325W variant) in soluble forms. In order to obtain soluble CTDs in sufficient amounts, the CTDs were expressed fused to a tag consisting of polyhistidine (His)-maltose binding protein (MBP)-tobacco etch virus protease recognition site (TEV) at the N-terminus (**a**). The His-MBP-TEV tagged CTDs were expressed in their soluble forms under the control of T7 promoter (inducible with 1 mM IPTG) in *E. coli* BL21 (DE3) cells. Protein samples of the His-MBP-TEV tagged CTDs (the WT (**b**) and the R325W variant (**c**) collected during different steps of purification (the Ni-NTA affinity and amylose affinity chromatography) were analyzed on the SDS-PAGE. Lane 1: *E. coli* BL21 (DE3) cell lysate, lane 2: unbound fraction from the Ni-NTA affinity column, lanes 3 and 4: second and last fractions collected during stringent washing with 30 mM imidazole, lanes 5 and 6: purified protein fractions eluted using 100 mM imidazole from Ni-NTA column, and lane 7: the Ni-NTA affinity purified protein further polished using amylose affinity chromatography. Lane M represents protein markers of known sizes.

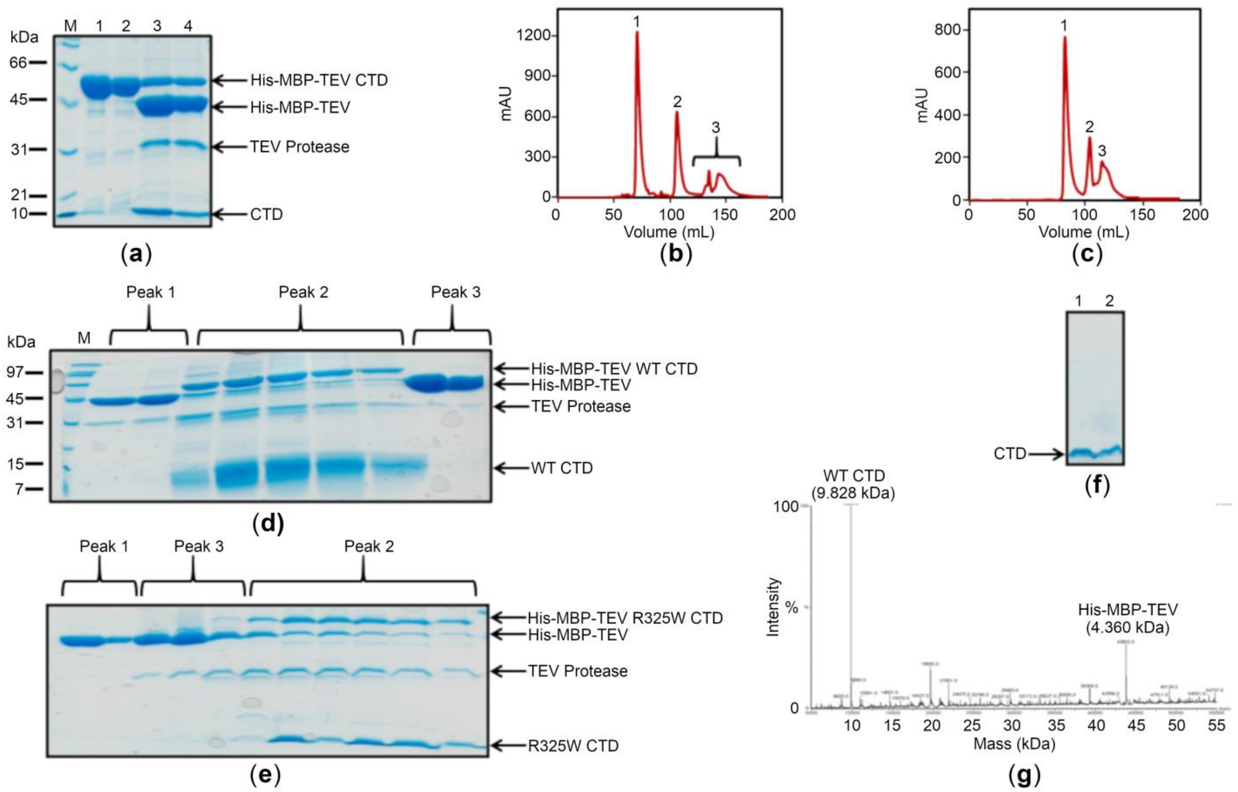

Figure S2. Purification of soluble CTDs in native forms and their analysis by SDS-PAGE. The Ni-NTA resins were used to purify the His-MBP-TEV WT CTD (panel **a**, lane 1) and the His-MBP-TEV R325W CTD (panel **a**, lane 2) through affinity chromatography. The N-terminal His-MBP-TEV tag was removed from the recombinant WT CTD (panel **a**, lane 3) and the recombinant R325W CTD (panel **a**, lane 4) using TEV protease. Ion-exchange chromatography was performed to separate the TEV-cleaved products of the His-MBP-TEV WT CTD (**b**) and the His-MBP-TEV R325W CTD (**c**) using Q HP columns (GE Healthcare UK). Fractions from peaks 1, 2 and 3 were analyzed by SDS-PAGE and showed uncleaved His-MBP-TEV WT CTD (**d**), uncleaved His-MBP-TEV R325W CTD (**e**), cleaved His-MBP-TEV tag (panels **d** and **e**), TEV protease (panels **d** and **e**), cleaved WT CTD (**d**), and cleaved R325W CTD (**e**). The cleaved CTDs from peak 2 (panels **d** and **e**) were separated from remaining oligohistidine and MBP tagged proteins through reverse affinity chromatography using Ni-NTA and amylose resins. Thus, soluble forms of the WT CTD (panel **f**, lane 1) and the R325W CTD (panel **f**, lane 2) were obtained. Lane M represents protein markers of known sizes. The cleaved His-MBP-TEV tag and the native WT CTD were further confirmed by mass spectrometry (**g**).

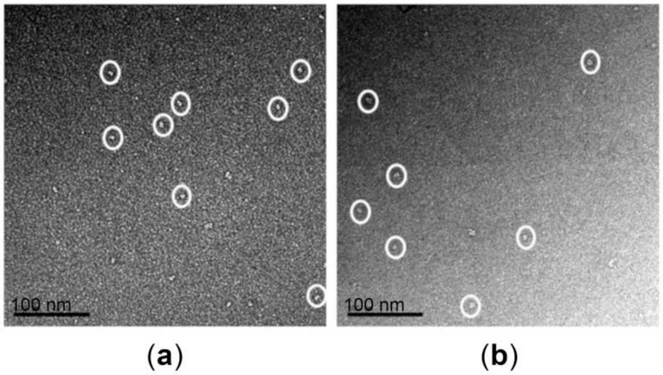

Figure S3. Analysis of purified CTDs by electron microscopy (EM). The EM analysis shows that the WT CTD (**a**) and the R325W CTD (**b**) are monodisperse (circled), and devoid of aggregates in solution.
